# The Mental Health Impact of the COVID-19 Pandemic Among Physicians, Nurses, and Other Health Care Providers in Alberta: Cross-sectional Survey

**DOI:** 10.2196/27469

**Published:** 2022-03-09

**Authors:** Hany El Gindi, Reham Shalaby, April Gusnowski, Wesley Vuong, Shireen Surood, Marianne Hrabok, Andrew J Greenshaw, Vincent Agyapong

**Affiliations:** 1 Critical Care Medicine Department King Abdul-Aziz Hospital Jeddah Saudi Arabia; 2 Department of Psychiatry University of Alberta Edmonton, AB Canada; 3 Addiction and Mental Health Alberta Health Services Edmonton, AB Canada; 4 Department of Psychiatry Cumming School of Medicine University of Calgary Calgary, AB Canada

**Keywords:** COVID-19, health care worker, mobile technology, Text4Hope, anxiety, depression, stress, pandemic, e-mental health, mental health, impact, physician, nurse, Canada

## Abstract

**Background:**

During the COVID-19 pandemic, threats to mental health, psychological safety, and well-being are evident, particularly among the first responders and the health care staff.

**Objective:**

This study aims to examine the prevalence and potential predictors of the likely stress, generalized anxiety disorder, and major depressive disorder among health care workers (HCWs).

**Methods:**

A cross-sectional survey was used through a survey link sent to gather demographic information and responses on several self-report scales, including the Perceived Stress Scale, the Generalized Anxiety Disorder 7-item scale, and the Patient Health Questionnaire-9 among HCWs enrolled in the Text4Hope program.

**Results:**

The result from this study suggests that during the COVID-19 pandemic, HCWs reported a high likelihood of moderate-to-high perceived stress (n=840, 81.2%), moderate-to-severe anxiety (n=369, 38.6%), and depression (n=317, 32.7%) symptoms. Nurses and other HCWs were significantly more likely to report depressive symptoms compared to physicians (F(2, 159.47)=15.89, 95% CI –5.05 to –2.04). Younger age groups of HCWs (≤30 years) were more prone to report likely stress, anxiety, and depressive symptoms compared to HCWs 41-50 and >50 years old (odds ratio [OR] 1.82-3.03). Similarly, females and those who reported a lack of social support (separated/divorced and single) among HCWs had a higher likelihood to report likely stress and depressive symptoms, respectively (OR 1.8 and 1.6, respectively).

**Conclusions:**

This cross-sectional study explored a high level of mental health burdens during the COVID-19 pandemic among HCWs in Alberta. Levels of psychological symptoms were more noticeable in the female gender and the nursing profession.

## Introduction

### Background

During pandemics, an exponential increase in the demand for health care services takes place. Several factors contribute to increased physical and mental health strain for health care workers (HCWs). This can include long work shifts, a lack of personal protective equipment, and limited resources to care for patients [1,2]. With a lack of PPE, HCWs can feel unprepared to deal with unknown viruses and bacteria. In addition, with pandemic demand–induced limits on resources, such as ventilators or general medical supplies, it can be difficult to care for patients.

In November 2019, a novel coronavirus disease caused by SARS-CoV-2, called COVID-19, was first reported in Wuhan, China. The disease rapidly spread throughout China and prevailed worldwide, resulting in a global health emergency [1]. On March 11, 2020, the World Health Organization declared the COVID-19 outbreak as a global pandemic [2].

Alberta Health Services (AHS) defines health care professionals/providers as “individuals that work in the health field and can include doctors, nurses (RNs, LPNs), dentists, psychologists, physiotherapists, pharmacists, and dieticians, etc” [3]. The nursing staff constitutes the largest health profession in Canada, followed by physicians and other regulated HCWs [4]. As of July 2020, the Canadian Institute for Health Information (CIHI) reported that 19.4% of all confirmed infections of COVID-19 cases were among HCWs in Canada, and in the province of Alberta, it was 8.8% [5]. HCWs are exposed to mental health stresses due to the nature of their work that usually involves trauma and vulnerability [6].

During the 2003 SARS outbreak, HCWs expressed immediate psychological distress manifested as fear and anxiety that exhibited a relative decrease during the early phases of the epidemic [1]. However, depression and posttraumatic stress symptoms emerged later during the epidemic and lasted for longer periods, affecting the long-term mental well-being of HCWs [1]. A greater psychological impact was coupled with the higher risk of exposure to the virus, especially among frontliners, who usually face both heavy workloads and a higher risk of infection [7,8]. One year after the SARS outbreak, HCWs who were in close contact with infected patients or virus material had elevated levels of stress, depression, and anxiety [9].

One study examining the psychological effects of COVID-19 on HCWs in Italy found that depression and posttraumatic stress symptoms are higher in HCWs caring for patients in COVID-19 wards compared to other HCWs caring for patients in other units [10].

Additionally, emotional impacts in terms of contagion fear and infecting loved ones, uncertainty, and stigma were documented among health care staff [11], leading to isolation from their families, changing routines, and a narrowing down of their social support network [12]. During the pandemic of COVID-19, health care professionals declared 5 main requests from their institutions [10]: “hear me, protect me, prepare me, support me, and care for me” [7]. Burnout is also an ongoing problem among physicians and other HCWs during the COVID-19 pandemic. It is characterized by “emotional exhaustion, depersonalization, and a feeling of low personal accomplishment” and attributed to many factors, including heavy workloads, that may exacerbate burnout and negatively impact the overall productivity of the health care system [13,14]. HCWs are predisposed to “moral injury” during the current pandemic, a term that can be defined as *the psychological distress that results from actions, or the lack of them, which violate someone’s moral or ethical code*. Although it is not a mental health condition, people with moral injury are more likely to experience negative thoughts about themselves or others, together with intense feelings of shame, guilt, or disgust [15].

During the COVID-19 pandemic, several studies have examined the impacts of the pandemic upon the psychological health and mental well-being of the general population. A number of studies have examined such impacts among HCWs, with many interesting findings; for example, the frontliners and those who have experienced physical symptoms, such as headache, throat pain, and lethargy are at more risk of developing stress, anxiety, and depressive symptoms during the pandemic compared to the other comparative groups [16,17].

In a systematic review assessing the impact of COVID-19 on HCWs’ mental health, the authors found that the prevalence of anxiety was estimated between 9 and 90% (median 24%) and depression between 5 and 51% (median 21%) [18]. In another systematic review and meta-analysis, Pappa et al [19] examined 13 research studies to compute the prevalence of anxiety, depression, and insomnia during the current pandemic. The authors reported 23.2% for anxiety and 22.8% for depression. Additionally, discrepancies of the prevalence or severity of such symptoms were reported among different demographic and occupational groups, such as in nurses, women, and frontliners who were usually reported to have more severe symptoms compared to other HCWs [19,20].

### Aim and Objectives

This study aims to examine the psychological impacts of the COVID-19 pandemic among different groups of HCWs who were enrolled in the Text4Hope program.

The primary objectives are:

- Studying the demographic characteristics and the prevalence and mean scores of perceived stress, likely major depressive disorder (MDD), and likely generalized anxiety disorder (GAD) among HCWs.
- Studying the predictors of developing likely stress, MDD, and GAD among HCWs.

## Methods

### Study Design

A cross-sectional survey was used to explore mean differences in perceived stress, anxiety, and depression symptom scores of HCWs enrolled in Text4Hope.

### Ethics

The authors assert that all procedures contributing to this work comply with the ethical standards of the relevant national and institutional committee on human experimentation and with the Declaration of Helsinki of 1975, as revised in 2008. Participant consent was implied by submission of the subscribers’ survey responses. The University of Alberta Health Research Ethics Board provided ethics approval for this research (Pro00086163).

### Recruitment

The study recruitment procedures and sample size estimations are described in a published study protocol [21]. The study participants are subscribers to the Text4Hope program, a daily supportive text message service, launched by the AHS on March 23, 2020, to help Albertans cope with the mental health effects of the COVID-19 pandemic. An online survey link was sent to subscribers who were enrolled during March 23 to May 2, 2020. Subscribers receive free daily supportive text messages, which are cognitive behavioral therapy–based messages created by a team of mental health professionals [22]. In addition to demographic information, clinical characteristics were assessed using self-report scales, including the Perceived Stress Scale (PSS-10; for moderate-to-high stress, PSS≥14) [23], the Generalized Anxiety Disorder 7-item (GAD-7) scale (for likely GAD, GAD-7≥10) [24], and the Patient Health Questionnaire-9 (PHQ-9; for likely MDD, PHQ-9≥10) [25]. The PSS-10 is a validated 10-item questionnaire used to assess the self-reported level of stress in the previous 1 month by assessing thoughts and feelings. Each item on the scale is scored from 0 (never) to 5 (very often). Higher scores on the scale denote higher levels of stress. The GAD-7 is a validated 7-item questionnaire used to assess the self-reported levels of anxiety in respondents in the 2 weeks prior to assessment. It is based on the *Diagnostic and Statistical Manual of Mental Disorders, Fourth Edition, Text Revision* (DSM-IV-TR) symptoms of anxiety. Each item on the scale is scored from 0 (not at all) to 4 (nearly every day). The PHQ-9 is a 9-item validated instrument used to diagnose and measure the severity of depression in general medical and mental health settings; it is the major depression module of the full Patient Health Questionnaire (PHQ). Each of the 9 items on the questionnaire is scored from 0 (not at all) to 3 (nearly every day). It may be used to plan and monitor treatment of depression. Participant consent was implied by submission of the subscribers’ survey responses.

### Data Analysis

Data analysis was undertaken using the IBM Statistical Package for Social Sciences (SPSS) Statistics for Windows, version 25 [26]. Descriptive analysis illustrated the differences between the 3 categories of HCWs (physicians, nurses, and other) by their sociodemographic characteristics (ie, gender, age, ethnicity, education, relationship status, housing status, and self-isolation/quarantine status) and examined the prevalence of clinical characteristics (ie, moderate/high stress, likely GAD, likely MDD) among the 3 HCW groups.

One-way ANOVA with 2-tailed significance (*P*<.05) was performed to assess the statistical differences of mean scores on the PSS-10, GAD-7, and PHQ-9 among HCW groups. For variables that did not violate the assumptions of homogeneity of variance in the mean scores on the ANOVA test, we performed a Tukey posthoc test. For variables that violated the homogeneity of variance assumption, we used the nonparametric Welch F test and Games-Howell posthoc tests.

To examine potential predictors of the self-reported clinically meaningful symptoms of moderate-to-high perceived stress, likely GAD, and likely MDD, we entered all demographic predictors and self-isolation, along with HCW type, into a multivariate logistic regression model. Correlation analysis was performed before logistic regression analysis to rule out strong correlations among predictor variables. Odds ratios (ORs) from the binary logistic regression analysis were examined to determine the association between HCW type and the likelihood of respondents self-reporting symptoms of moderate-to-high stress, likely GAD, and likely MDD, controlling for the other variables in the model.

## Results

### Participant Characteristics

Of the 8267 subscribers who responded to the Text4Hope online survey in the first 6 weeks, 1414 (17.1%) self-identified as HCWs, while 1096 (13.3%) provided their specific occupation type. Of these 1096, 63 (5.7%) were physicians, 355 (32.4%) nurses, and 678 (61.9%) other HCWs (eg, occupational therapist, psychologist, dietitian, pharmacist, and first responder). The demographic and clinical characteristics of the respondents are shown in Table 1.

As presented in Table 1, most of the 1096 respondents were female (n=1006, 91.8%), aged 31-40 years (n=330, 30.1%), and Caucasian (n=919, 83.9%); had postsecondary education (n=1066, 97.3%); were married, cohabiting, or partnered (n=852, 77.7%); and owned their own home (n=836, 76.3%). Except for a few variables, nurses represented the highest percentage of these responses.

**Table 1:** Distribution of the demographic and clinical characteristics by HCW^a^ type.

Variables		Physicians (N=63)	Nurses (N=355)	Other (N=678)	Total (N=1096)^b^
**Gender, n (%)**					
	Male	17 (27.0)	10 (2.8)	55 (8.1)	82 (7.5)
	Female	46 (73.0)	343 (96.9)	617 (91.1)	1006 (92.0)
	Other	0	1 (0.3)	5 (0.7)	6 (0.5)
**Age (years), n (%)**					
	≤30	13 (21.0)	5 (16.8)	90 (13.5)	162 (15.0)
	31-40	9 (14.5)	107 (30.4)	214 (32.2)	330 (30.6)
	41-50	19 (30.6)	78 (22.2)	193 (29.0)	290 (26.9)
	>50	21 (33.9)	108 (30.7)	168 (25.3)	297 (27.5)
**Ethnicity, n (%)**					
	Caucasian	48 (76.2)	309 (87.0)	562 (83.0)	919 (83.9)
	Indigenous	1 (1.6)	6 (1.7)	24 (3.5)	31 (2.8)
	Asian	10 (15.9)	15 (4.2)	44 (6.5)	69 (6.3)
	Other	4 (6.3)	25 (7.0)	47 (6.9)	76 (6.9)
**Education, n (%)**					
	Less than high school diploma	0	1 (0.3)	2 (0.3)	3 (0.3)
	High school diploma	0	1 (0.3)	16 (2.4)	17 (1.6)
	Postsecondary education	63 (100.0)	351 (99.2)	652 (96.4)	1066 (97.5)
	Other education	0	1 (0.3)	6 (0.9)	7 (0.6)
**Relationship status, n (%)**					
	Married/cohabiting/partnered	51 (81.0)	291 (82.0)	510 (75.3)	852 (77.8)
	Separated/divorced	3 (4.8)	24 (6.8)	61 (9.0)	88 (8.0)
	Widowed	0	3 (0.8)	3 (0.4)	6 (0.5)
	Single	9 (14.3)	37 (10.4)	98 (14.5)	144 (13.2)
	Other	0	0	5 (0.7)	5 (0.5)
**Housing status, n (%)**					
	Own home	49 (77.8)	287 (80.8)	500 (73.9)	836 (76.3)
	Living with family	4 (6.3)	12 (3.4)	24 (3.5)	40 (3.7)
	Renting	10 (15.9)	55 (15.5)	147 (21.7)	212 (19.4)
	Other	0	1 (0.3)	6 (0.9)	7 (0.6)
**Self-isolate/quarantine, n (%)**					
	No	41 (65.1)	273 (77.1)	540 (80.1)	854 (78.3)
	Yes	22 (34.9)	81 (22.9)	134 (19.9)	237 (21.7)
**Symptoms, n (%)^c^**					
	Perceived stress (moderate to high^d^)	41 (68.3)	279 (82.5)	520 (81.6)	840 (81.2)
	Likely GAD^e^	20 (36.4)	116 (37.4)	233 (39.4)	369 (38.6)
	Likely MDD^f^	9 (16.1)	99 (31.3)	209 (35.1)	317 (32.7)
**Clinical condition severity, mean (SD)**					
	PSS-10^g^ ANOVA F_(2, 1032)_=1.13, *P*=.32	18.07 (7.25)	19.25 (6.27)	19.35 (6.28)	19.25 (6.34)
	GAD-7^h^ ANOVA F_(2, 953)_=0.80, *P*=.45	4.46 (4.36)	7.42 (5.17)	8.01 (5.72)	7.61 (5.53)
	PHQ-9^i^ Welch statistics F(2, 159.47)=15.89, *P*<.001	7.58 (5.25)	8.18 (5.32)	8.46 (5.58)	8.32 (5.48)

^a^HCW: health care worker.

^b^Data were reported according to the provided responses. The total percentages may not add up to 100%.

^c^Multiple responses were possible.

^d^Moderate-to-high stress was defined as PSS-10 score of ≥14.

^e^Likely GAD was defined as a GAD-7 score of ≥10.

^f^Likely MDD was defined as a PHQ-9 score of ≥10.

^g^PSS-10: Perceived Stress Scale.

^h^GAD-7: Generalized Anxiety Disorder 7-item.

^i^PHQ-9: Patient Health Questionnaire-9.

Among the 1096 respondents, 237 (21.7%) reported having self-isolated/quarantined during the pandemic time. Among those who reported their clinical symptoms, most respondents who self-reported moderate-to-high perceived stress were nurses (279/338, 82.5%), while those who self-reported likely GAD and likely MDD were other HCWs (233/591, 39.4%) and (209/596, 35.1%), respectively.

The mean scores for all HCWs were 19.25 (SD 6.34, n=1035) on the PSS-10 scale, 8.32 (SD 5.48, n=956) on the GAD-7 scale, and 7.61 (SD 5.53, n=968) on the PHQ-9 scale.

One-way ANOVA revealed a significant difference among the 3 groups of HCWs only in terms of their PHQ-9 mean scores. The Welch test and Games-Howell posthoc tests showed that physicians (mean=4.46, SD 4.36) scored significantly lower on the PHQ-9 scale compared to nurses (mean=7.42, SD 5.17, 95% CI –4.51 to –1.4, *P*<.01) and to other HCWs (mean=8.01, SD 5.72, 95% CI –5.05 to –2.04, *P*<.001).

### Logistic Regression

Spearman correlation analysis revealed no high collinearity among the suggested variables (r_s_<0.4), so all the variables were entered into the multivariate regression model.

Table 2 presents data indicating that for moderate-to-high likely stress, the model containing the 8 predictors was statistically significant (χ^2^_21_=68.76, *P*<.001). The model explained between 6.6% (Cox and Snell R^2^) and 10.6% (Nagelkerke R^2^) of the variance and correctly classified 80.8% of all cases. The type of HCWs did not significantly contribute to the likely stress model among all the HCWs. Controlling for all other factors in the model, age categories made a unique statistical contribution (Wald=34.24, *P*<.001) to the likelihood that a respondent presented with moderate-to-high stress.

**Table 2:** Logistic regression predicting likelihood for the respondents among HCWs^a^ to present with moderate-to-high stress.

Variables		B	SE	Wald	*df*	*P* value^b^	OR^c^ (95% CI)
**Gender**							
	Male	—^d^	—	5.451	2	.07	—
	Female	0.683	0.293	5.442	1	*.02*	1.980 (1.115-3.515)
	Other	0.714	1.211	0.347	1	.56	2.041 (0.190-21.907)
**Age (years)**							
	≤30	—	—	34.244	3	<*.001*	—
	31-40	0.567	0.330	2.956	1	.09	1.763 (0.924-3.366)
	41-50	–0.310	0.313	0.980	1	.32	0.733 (0.397-1.355)
	>50	–0.845	0.309	7.469	1	*.01*	0.429 (0.234-0.787)
**Ethnicity**							
	Caucasian	—	—	0.548	3	.91	—
	Indigenous	0.358	.644	0.309	1	.58	1.430 (0.405-5.052)
	Asian	–0.160	.368	0.189	1	.66	0.852 (0.414-1.753)
	Other	–0.070	.337	0.043	1	.84	0.933 (0.482-1.805)
**Education**							
	Less than high school diploma	—	—	1.442	3	.69	—
	High school diploma	1.430	1.661	0.741	1	.39	4.177 (0.161-108.342)
	Postsecondary education	0.751	1.282	0.343	1	.56	2.118 (0.172-26.144)
	Other education	–.043	1.597	0.001	1	.98	0.958 (0.042-21.934)
**Relationship status**							
	Married/cohabiting/partnered	—	—	1.239	4	.87	—
	Separated/divorced	–.090	0.300	0.089	1	.77	0.914 (0.508-1.646)
	Widowed	–.362	0.885	0.167	1	.68	0.696 (0.123-3.948)
	Single	.253	0.297	0.730	1	.39	1.288 (0.720-2.304)
	Other	–.444	1.272	0.122	1	.73	0.641 (0.053-7.758)
**Housing status**							
	Own home	—	—	2.175	3	.54	—
	Living with family	.743	0.656	1.285	1	.26	2.103 (0.582-7.599)
	Renting	.296	0.265	1.244	1	.27	1.344 (0.799-2.261)
	Other	20.278	15,888.453	0	1	.99	640,763,554.13 (0 to upper level)
**Self-isolate/quarantine**							
	No	—	—	—	—	—	—
	Yes	–.108	0.204	0.281	1	.59	0.898 (0.602-1.339)
**HCW type**							
	Physician	—	—	2.874	2	.24	—
	Nurses	.573	0.339	2.864	1	.09	1.774 (0.913-3.446)
	Other	.474	0.321	2.180	1	.14	1.606 (0.856-3.012)
**Constant**		–.200	1.347	.022	1	.88	0.819 (—)

^a^HCW: health care worker.

^b^Significant *P* values are italicized.

^c^OR: odds ratio.

^d^—: Not applicable.

HCWs who were >50 years old were less likely to report moderate-to-high stress during the COVID-19 pandemic compared to respondents who were ≤30 years old, when all other variables in the model were controlled (OR 0.43, 95% CI 0.23-0.79). Although the gender variable did not significantly contribute to the model, females were almost 2 times more likely to report moderate-to-high stress during the COVID-19 pandemic compared to males, when controlling for other variables (OR 2.0, 95% CI 1.12-3.52).

The data in Table 3 indicate that for likely GAD, the 8-predictor model was statistically significant (χ^2^_21_=70.82, *P*<.001), explaining between 7.3% (Cox and Snell R^2^) and 9.9% (Nagelkerke R^2^) of the variance and correctly classified 64.9% of all cases. The type of HCWs did not significantly contribute to likely GAD among all the HCWs. Likewise, age categories made a unique statistical contribution (Wald=41.85, *P*<.001) to the probability that a respondent presented with likely GAD, after controlling for all other factors in this model. HCWs who were 41-50 years old and those who were >50 years old had a lower probability of reporting likely GAD symptoms during the COVID-19 pandemic compared to those who were ≤30 years old (OR 0.41, 95% CI 0.26-0.66 vs OR 0.33, 95% CI 0.2-0.53).

**Table 3:** Logistic regression predicting likelihood for the respondents among HCWs^a^ to present with likely GAD^b^.

Variables		B	SE	Wald	*df*	*P* value^c^	OR^d^ (95% CI)
**Gender**							
	Male	—^e^	4.250	2	2	.12	—
	Female	0.468	2.497	1	1	.11	1.597 (0.894-2.852)
	Other	1.657	2.898	1	1	.09	5.243 (0.778-35.319)
**Age (years)**							
	≤30	—	—	41.852	3	*<.001*	—
	31-40	–0.045	0.224	0.040	1	.84	0.956 (0.616-1.483)
	41-50	–0.887	0.243	13.344	1	*<.001*	0.412 (0.256-0.663)
	>50	–1.121	0.250	20.131	1	*<.001*	0.326 (0.200-0.532)
**Ethnicity**							
	Caucasian	—	—	2.910	3	.41	—
	Indigenous	0.453	0.421	1.161	1	.28	1.574 (0.690-3.591)
	Asian	–0.247	0.312	0.625	1	.43	0.781 (0.424-1.441)
	Other	0.288	0.291	0.984	1	.32	1.334 (0.755-2.359)
**Education**							
	Less than high school diploma	—	—	2.004	3	.57	—
	High school diploma	–0.585	1.444	0.164	1	.69	0.557 (0.033--9.442)
	Postsecondary education	–1.194	1.285	0.864	1	.35	0.303 (0.024-3.760)
	Other education	–1.790	1.669	1.151	1	.28	0.167 (0.006-4.394)
**Relationship status**							
	Married/cohabiting/partnered	—	—	2.865	4	.58	—
	Separated/divorced	0.148	0.274	0.293	1	.59	1.160 (0.678-1.984)
	Widowed	–0.673	1.110	0.367	1	.55	0.510 (0.058-4.498)
	Single	0.293	0.220	1.768	1	.18	1.340 (0.870-2.064)
	Other	–0.691	1.072	0.416	1	.52	0.501 (0.061-4.095)
**Housing status**							
	Own home	—	—	3.756	3	.29	—
	Living with family	0.027	0.412	0.004	1	.95	1.028 (0.458-2.305)
	Renting	0.028	0.196	0.020	1	.89	1.028 (0.699-1.510)
	Other	2.323	1.200	3.748	1	.05	10.210 (0.972-107.273)
**Self-isolate/quarantine**							
	No	—	—	—	—	—	—
	Yes	–0.236	0.172	1.879	1	.17	0.790 (0.563-1.107)
**HCW type**							
	Physician	—	—	0.400	2	.82	—
	Nurses	–0.113	0.330	0.117	1	.73	0.893 (0.467-1.706)
	Other	–0.019	0.318	0.003	1	.95	0.981 (0.526-1.831)
**Constant**		0.848	1.351	0.394	1	.53	2.334 (—)

^a^HCW: health care worker.

^b^GAD: generalized anxiety disorder.

^c^Significant *P* values are italicized.

^d^OR: odds ratio.

^e^—: Not applicable.

The data displayed in Table 4 indicate that for likely MDD, the model containing the 8 predictors was statistically significant (χ^2^_21_=69.14, *P*<.001). The model explained between 7.0% (Cox and Snell R^2^) and 9.8% (Nagelkerke R^2^) of the variance and correctly classified 68% of all cases. After controlling for all other factors in the model, the type of HCWs made a unique statistical contribution (Wald=6.1, *P*=.05) to the likelihood that a respondent presented with moderate-to-high MDD. Nurses and other HCWs exhibited a 2 times greater probability of presenting with likely MDD during the pandemic compared to physicians (OR 2.32, 95% CI 1.06-5.10 vs OR 2.60, 95% CI 1.20-5.58). The variable of age categories made a unique statistical contribution (Wald=24.54, *P*<.001) to the probability that a respondent presented with likely MDD. Like the previous (GAD) model, HCWs who were 41-50 and >50 years old had a lower OR to report likely GAD symptoms during the COVID-19 pandemic compared to those ≤30 years old (OR 0.55, 95% CI 0.34-0.90 vs OR 0.39, 95% CI 0.24-0.65). Although the relationship status variable did not contribute significantly to the model, separated/divorced and single respondents reported a higher likelihood of MDD compared to married/cohabiting/partnered respondents when controlling for other variables (OR 1.80, 95% CI 1.04-3.02 vs OR 1.60, 95% CI 1.07-2.52).

From the 3 models collectively, there was a trend for age, whereby HCWs who were below 40 years old appeared more likely to exhibit psychological clinical symptoms compared to older participants.

**Table 4:** Logistic regression predicting likelihood for the respondents among HCWs^a^ to present with likely MDDb.

Variables		B	SE	Wald	*df*	*P* value^c^	OR^d^ (95% CI)
**Gender**							
	Male	—^e^	—	0.619	2	.73	—
	Female	0.031	0.294	0.011	1	.92	1.032
	Other	0.714	0.918	0.606	1	.44	2.042
**Age (years)**							
	≤30	—	—	24.539	3	*<.001*	—
	31-40	–0.025	0.227	0.012	1	.91	0.975
	41-50	–0.590	0.247	5.703	1	*.02*	0.554
	>50	–0.934	0.258	13.111	1	*<.001*	0.393
**Ethnicity**							
	Caucasian	—	—	1.282	3	.73	—
	Indigenous	0.046	0.427	0.011	1	.92	1.047
	Asian	–0.075	0.321	0.054	1	.82	0.928
	Other	0.319	0.295	1.168	1	.28	1.376
**Education**							
	Less than high school diploma	—	—	3.471	3	.32	—
	High school diploma	–0.677	1.406	0.232	1	.63	0.508
	Postsecondary education	–1.500	1.272	1.390	1	.24	0.223
	Other education	–0.950	1.640	0.336	1	.56	0.387
**Relationship status**							
	Married/cohabiting/partnered	—	—	8.863	4	.07	—
	Separated/divorced	0.574	0.271	4.493	1	*.03*	1.776
	Widowed	–0.273	1.109	0.060	1	.81	0.761
	Single	0.495	0.219	5.107	1	*.02*	1.640
	Other	–0.537	1.055	0.259	1	.61	0.584
**Housing status**							
	Own home	—	—	4.775	3	.19	—
	Living with family	.356	.413	0.744	1	.39	1.428
	Renting	.225	.195	1.326	1	.25	1.252
	Other	2.195	1.201	3.340	1	.07	8.980
**Self-isolate/quarantine**							
	No	—	—	—	—	—	—
	Yes	–0.114	0.177	0.418	1	0.52	0.892
**HCW type**							
	Physician	—	—	6.076	2	*.05*	—
	Nurses	0.843	0.402	4.396	1	*.04*	2.322
	Other	0.952	0.391	5.932	1	*.02*	2.592
**Constant**		0.095	1.358	0.005	1	.94	1.099 (—)

^a^HCW: health care worker.

^b^MDD: major depressive disorder.

^c^Significant *P* values are italicized.

^d^OR: odds ratio.

^e^—: Not applicable.

## Discussion

### Principal Findings

Using self-reported data from the Text4Hope service, this study illustrates the different mental health impacts of the COVID-19 pandemic on HCWs, including physicians, nurses, and other HCWs. The study suggests that more than 4 in 5 HCWs expressed a likelihood of reporting moderate-to-high perceived stress rates, while around a third expressed a likelihood of reporting moderate-to-severe anxiety and depressive symptoms during the COVID-19 pandemic. In Muller et al’s [18] systematic review, it was determined that the prevalence of mental health distress ranged from 7% to 97%, with a median of 37%. In our study, the prevalence of GAD and depression in HCWs was 38.6% and 32.7%, respectively, which was comparable to the medians reported in the aforementioned 2 systematic reviews [18,19].

In a survey of health care providers in Wuhan, the number of frontline health care providers with depressive symptoms was estimated to be 50.4%, with correlates of nursing profession and female gender [20]. This higher figure compared with the prevalence of depression in our study may be explained by the demographics of the population that study focused on, as most participants were female, nurses, of lower ages, and with a junior technical title that could be considered as elevating COVID-19 exposure risk factors for the development of depressive symptoms [20,27].

Physicians had the lowest prevalence of GAD and depression in this study (20/63 [36.4%] and 9/63 [16.1%], respectively), followed by nurses (116/355 [37.4%] and 99/355 [31.3%], respectively) and was highest in other HCW groups (233/678 [39.4%] and 209/678 [35.1%], respectively). Pappa et al [19] noted similar trends in the prevalence of depression and anxiety between physicians and nurses in their systematic review. Physicians had a lower prevalence of depression (25.4%) when compared with nurses (30.3%), and this was the case for GAD that was found in 21.7% of physicians, while 25.8% of nurses were affected [19].

Compared to the frontline physicians, first responder nurses are more likely to develop behavioral disengagement during similar epidemics, a result highly associated with aggravating levels of depression [28,29]. This may be due to stress and the fear of contracting the infection or spreading it to their families, along with the perceived stigma and sense of uncertainty [20]. Consistent with our research, where nurses were found to be more likely to report moderate-to-severe likely stress symptoms compared to the physicians, in a study carried out in Wuhan, China, nurses were also reported to experience higher levels of different psychological impacts, such as distress, anxiety, depression, and insomnia, when compared to their physician counterparts during the COVID-19 pandemic [20].

Females comprised most of our survey respondents (1006/1096, 92%) and showed a higher likelihood to report stress, GAD, and depressive symptoms compared to their male counterparts. However, this was statistically significant only for likely stress symptoms. This was consistent with other studies. For example, after pooling the prevalence from 6 studies that reported complete data for anxiety symptoms based on gender during the COVID-19 pandemic, Pappa et al [19] reported a lower prevalence of anxiety in males (20.9%) compared to females (29.1%). Conversely, they noted that the prevalence of depression was higher in males (26.9%) than in females (20.3%) [19]. Several studies have demonstrated that being a woman, getting exposed to patients with SARS-CoV-2 infection, and worrying about being infected are the most common risk factors associated with increased mental health problems in HCWs during the pandemic [18].

Younger HCWs were generally at a higher risk of developing likely stress, anxiety, and depression compared to other groups. This finding is aligned with other previous findings that consistently report a higher risk among younger ages during the pandemic [27,30,31]. This may be attributed to the longer duration the younger generation usually spends focusing upon the data of the pandemic or to the lack of experiencing similar stressful situations in their few years of experience compared to older groups, who possibly have passed by similar experiences [27,30].

In our study, the lack of a confiding relationship (separated/divorced and single) appeared to be a risk factor for HCWs to self-report likely depression. It is likely that HCWs who are in confiding relationships have better social supports that those who are single, divorced, or widowed. This finding appears clearly in the literature, where social, family, and friend support is found to be the most commonly reported protective factor associated with a reduction in mental health problems among HCWs [18,32].

Not surprisingly, the younger age group of HCWs expressed a higher likelihood to show likely stress, GAD, and MDD symptoms compared to the other older groups. Additionally, the likelihood to report the 3 clinical conditions of focus in our study seemed to decrease with age, where HCWs who were ≤30 years old were at a higher risk of developing likely stress, GAD, and MDD during the pandemic compared to those who were 41-50 and >50 years old (OR 1.82-3.03). This aligns with previous research that reported the same finding among all subscribers of Text4Hope [27,33]. Relatively little experience and the lack of exposure to similar epidemics in the younger age group could predispose younger people to a greater likelihood of developing maladjustment and mental distress in this context [27,34].

Other researchers are examining COVID-19-related measurements among HCWs across Canada, including exposure to the virus and other mental health parameters [13]. Preliminary results from the other study suggests that there are high COVID-19 infection rates among HCWs and that physicians are more likely than other HCWs to develop mental health symptoms. These results differ from the findings of our study, which suggest that physicians are less likely to develop mental health symptoms during the COVID-19 pandemic compared to nurses and other HCWs. Observed differences between each study’s results could be attributed to differences in data collection periods (eg, early pandemic, during a wave), differences in COVID-19 positivity rates and R values, or even differences in the level of resource strain (eg, bed capacity, worker shortages) on the health care system. Although our data were collected during the early phase of the pandemic, when the health system was not strained, data from an ongoing Canadian study were collected at a later stage of the pandemic, when infection rates were higher and health care systems across Canada were under greater strain.

During the COVID-19 pandemic, some digital initiatives took place to mitigate psychological distress among health care professionals; these included a digital support package on psychological well-being provided for HCWs in the United Kingdom. The package was easily delivered and reached the target group, with a high rate of usage and accepted cost [7]. In a recent review of the literature demonstrating the mental health problems that HCWs are facing during the COVID-19 pandemic, the authors concluded that health authorities need to build multidisciplinary mental health teams in order to mitigate mental health and psychological consequences of the pandemic on both patients and HCWs. It was suggested that electronic media through web apps could be used for this purpose [6]. In Alberta, Canada, the AHS launched a supportive text message program (Text4Hope). The service aims to support the mental health of Albertans during the COVID-19 pandemic and is presented as a feasible and reliable medium for wide-scale data collection for epidemiological research, as illustrated by this report.

### Limitations

This study had several limitations. The data were obtained via an online request sent to all subscribers of the Text4Hope service, and there was a risk of selection bias, where the HCWs who responded may have been more interested in the service and not necessarily representative of all HCWs in Alberta. Females also showed overrepresentation in our cohort, which may limit the generalization of the provided results. Additionally, as self-report scales were used in this study and participants were not clinically assessed or confirmed, the results need to be interpreted carefully.

Finally, this was a cross-sectional study with no established baseline prevalence or a control group to compare with. It cannot draw any inferences about the impact of the pandemic on HCWs’ mental health; thus, the presented findings may need further similar studies across different timepoints into the pandemic to validate our results.

### Conclusion

This cross-sectional study explored the impact of the COVID-19 pandemic on the mental health and well-being of HCWs in Alberta. Overall, we highlighted the prevalence of the psychological distress symptoms in the early phase of the COVID-19 pandemic. Nurses were observed to be at high risk for developing stress, depression, and anxiety during the pandemic. Tracking such symptoms through digitally supported means is highly emphasized, particularly during pandemics where physical contact is not a viable option. Usually, digitally provided programs may yield high fidelity in relation to their acceptability and engagement [35]. Such services are impactful and cost-effective and fulfil essential social distancing requirements with remote delivery.

In the light of time progression and the development of the knowledge around the pandemic and the available vaccination, we aim to examine the changes in these symptoms and their timely progress among the health care providers after 1 year of the initiation of the Text4Hope service. From the literature, positive impacts of similar interventions were reported, in terms of reducing depressive symptoms and increasing the abstinence duration in alcohol use disorder, after 3 months of receiving daily supportive text messages in the community settings [36,37]. The availability of the texting service on most cell phones, and the lack of required software or app downloads to function, rendered text messaging services significantly advantageous over similar technologies (eg, email or messaging apps) [36].

## Abbreviations

AHS: Alberta Health Services
GAD: generalized anxiety disorder
GAD-7: Generalized Anxiety Disorder 7-item
HCW: health care worker
MDD: major depressive disorder
OR: odds ratio
PHQ-9: Patient Health Questionnaire-9
PSS-10: Perceived Stress Scale
